# Habitat Fragmentation Drives Plant Community Assembly Processes across Life Stages

**DOI:** 10.1371/journal.pone.0159572

**Published:** 2016-07-18

**Authors:** Guang Hu, Kenneth J. Feeley, Mingjian Yu

**Affiliations:** 1 College of Life Sciences, Zhejiang University, Hangzhou, Zhejiang, China; 2 Department of Landscape Architecture, School of Civil Engineering and Architecture, Zhejiang Sci-Tech University, Hangzhou, Zhejiang, China; 3 International Center of Tropical Botany, Department of Biological Sciences, Florida International University, Miami, Florida, United States of America; 4 Center for Tropical Plant Conservation, Fairchild Tropical Botanic Garden, Coral Gables, Florida, United States of America; Chinese Academy of Forestry, CHINA

## Abstract

Habitat fragmentation is one of the principal causes of biodiversity loss and hence understanding its impacts on community assembly and disassembly is an important topic in ecology. We studied the relationships between fragmentation and community assembly processes in the land-bridge island system of Thousand Island Lake in East China. We focused on the changes in species diversity and phylogenetic diversity that occurred between life stages of woody plants growing on these islands. The observed diversities were compared with the expected diversities from random null models to characterize assembly processes. Regression tree analysis was used to illustrate the relationships between island attributes and community assembly processes. We found that different assembly processes predominate in the seedlings-to-saplings life-stage transition (SS) vs. the saplings-to-trees transition (ST). Island area was the main attribute driving the assembly process in SS. In ST, island isolation was more important. Within a fragmented landscape, the factors driving community assembly processes were found to differ between life stage transitions. Environmental filtering had a strong effect on the seedlings-to-saplings life-stage transition. Habitat isolation and dispersal limitation influenced all plant life stages, but had a weaker effect on communities than area. These findings add to our understanding of the processes driving community assembly and species coexistence in the context of pervasive and widespread habitat loss and fragmentation.

## Introduction

The mechanisms determining how plant communities assemble have long been a focus of research in community ecology [1–7]. The question of which factors or processes determine community assembly and species co-existence has received a great deal of attention because of its relevance to the maintenance and conservation of biodiversity [4,8–10]. Studies conducted to date indicate that 1) environmental filtering, 2) selective immigration, 3) negative density dependence, 4) competition and 5) stochastic processes can all drive patterns of community assembly [1,5,11–13]. He *et al*. [10] suggested that the contributions of these mechanisms to community assembly can differ between taxa and across different life stages.

Species diversity is commonly used as an expedient means for evaluating the main assembly processes in plant communities since different mechanisms are expected to lead to specific shifts of diversity patterns. However, some studies have suggested that analyzing patterns of species diversity alone is not sufficient distinguish the main assembly processes [14] since this method ignores the potential effects of the interspecific associations on species composition. In more recent studies, phylogenetic diversity has been used to help reveal the mechanisms of species coexistence and community assembly [3,14–16] because phylogenetic diversity is linked to the functional and evolutionary differences between species [3]. Assuming phylogenetic conservatism, such that more closely related species are expected to occupy more similar niches [17–19], patterns of phylogenetic diversity may reflect the determinant ecological mechanisms in community assembly and thus may be a useful complement to species diversity.

**Fig 1:**
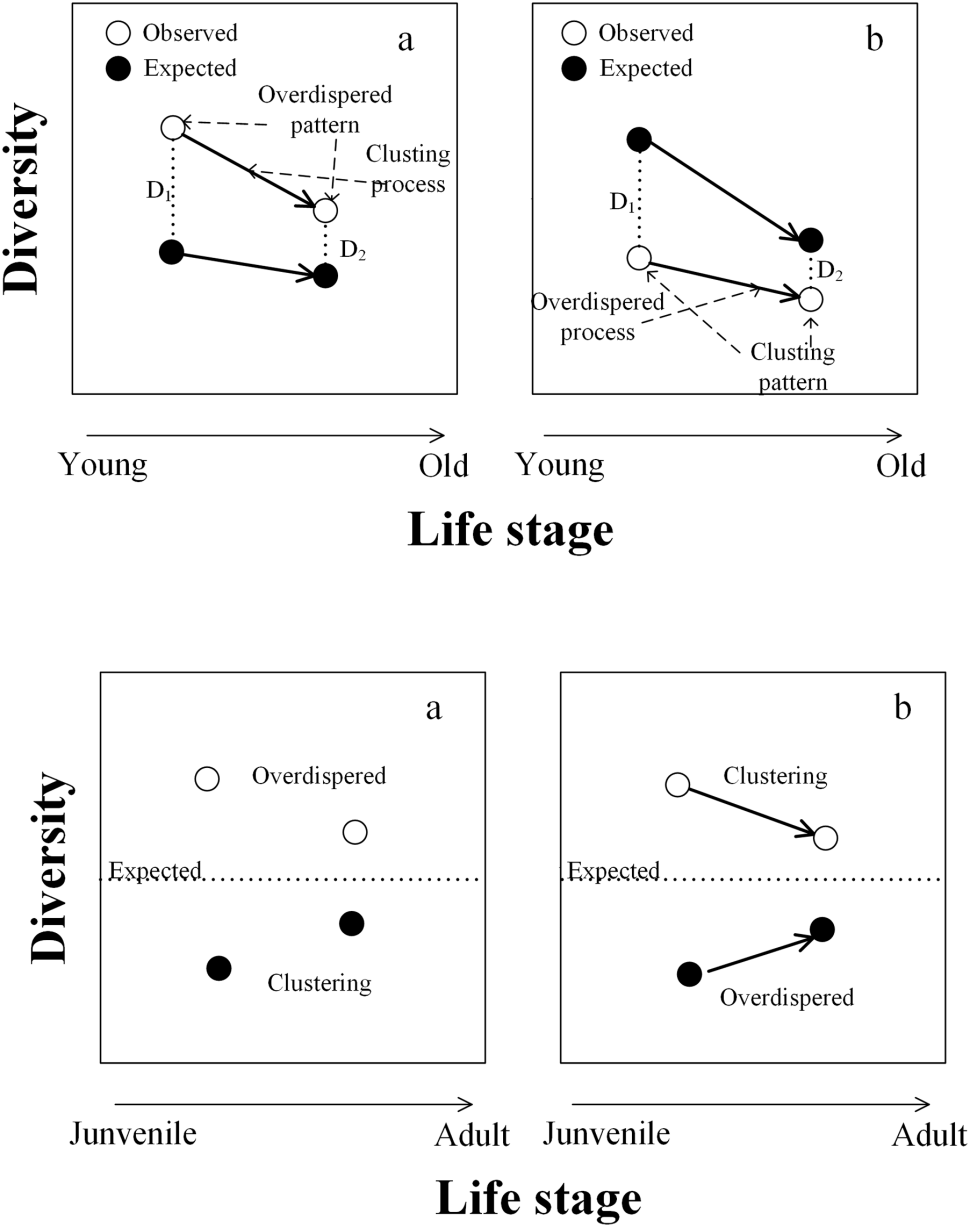
A conceptual graph of community assembly (a) without and (b) with the association across life stages. The open and closed points represent the diversity of two communities at different life stages, respectively. The arrows (in b) show the trend from one life stage to next was considered. The open points without association (a) are both greater than expected, which indicates an overdispersed assembly pattern according to Webb’s hypothesis (Webb *et al*., 2002). However, when we consider the association between life stages, the diversity with a decreasing trend from one stage to the next (open points in b), indicates clustering assembly process, regardless whether these values of these points are greater or less than expected. The reversed performances of closed points are also shown.

For plants, propagule dispersal, seedling establishment and fast growth are often thought to be the primary processes driving community assembly at the seedling and sapling stages [20,21], while competition, disturbance resistance and propagation are likely to be main processes driving community assembly at the adult stage [5,22–24]. Most previous studies on community assembly have only focused on a static state or one specific life stage (e.g., just seedlings), or have analyzed the different life stages independently without their association [15,25,26]. However, plant community assembly is actual a dynamic process that is better considered within a time series framework or across life stages (e.g. from seedlings to saplings). Overlooking the association across life stages may lead to different or misleading results (Fig 1). Thus, the shift of diversity from one life stage to next (Fig 1b), for example in comparisons of seedlings vs. saplings comparison (SS), or in comparisons of sapling to trees (ST), can potentially be used to more robustly evaluate community assembly processes. In the present study, we defined the assembly process as the change of assembly patterns across life stages, which is represented by variation of diversity metrics (Table 1). For example, environmental filtering is predicted to select for species with similar resource demands, thereby decreasing the functional and phylogenetic diversity through life stages. Competitive exclusion can also decreases species diversity, but in contrast to habitat filtering should lead to increasing phylogenetic of functional diversity.

**Table 1:** Predicted effects of different ecological processes on the shift of species and phylogenetic diversity and community assembly process from one life stage to next.

Ecological mechanism	Species level		Phylogenetic level	
	Diversity change	Assembly process	Diversity change*	Assembly process
Environmental filtering**	Decrease	Clustering	Decrease	Clustering
Dispersal limitation***	Decrease	Clustering	Decrease	Clustering
Density dependence	Increase	Overdispersed	Increase	Overdispersed
Interspecific competition	Decrease	Clustering	Increase	Overdispersed
Random process	No change	Random	No change	Random

* Assuming phylogenetic niche conservatism.

**Referring to island area and shape in our island system.

***Referring to isolation in our island system.

Most studies investigating the mechanisms of species coexistence focus on environmental and spatial attributes such as topography and soil fertility in a continuous habitat [8,25,27]. However, the effects of landscape variables on community assembly and species coexistence cannot be addressed in these studies because of lack of replication [28]. Furthermore, the influence of habitat loss and fragmentation on community assembly has also not been well tested with field data despite the predicted influences [29]. Habitat fragmentation is one of the most widespread and important threats to biodiversity. It can have instantaneous effects on population sizes, and the associated changes of landscape configuration (area, shape or isolation) can also have important long-term consequences for ecological patterns and processes [9,30] and biodiversity [31,32].

Previous studies that we have conducted on recently-formed land-bridge islands/fragments in the Thousand Island Lake (TIL) of China have shown that changes of habitat area, isolation, shape and other attributes significantly affected the distribution of plant species [30,32]. These changes of landscape configuration may also influence community assembly [33]. For example, Perdomo *et al*. [34] found that the connectivity of fragmented landscapes modified arthropod community assembly by influencing the dispersal and source pool of recolonizing fauna. Habitat fragmentation also affected the dynamics of parasites and pathogens leading to the re-assemblage of the community [35]. Considering the pervasive global occurrence of habitat fragmentation, it is necessary and urgent to understand the influences of fragmentation on community assembly processes.

In this study, we used a comprehensive approach analyzing shifts of species and phylogenetic diversity between different plant life stages to distinguish the mechanisms shaping community assembly and to test the influence of island attributes linking to habitat fragmentation on community assembly. We applied this approach to data collected for trees from 29 isolated land-bridge islands and surrounding mainland at TIL, China. We used data from three life stages (seedling, sapling and tree) and computed the change of species and phylogenetic diversity across life stages. We then evaluated the effects of island attributes on shaping community assembly. We specifically addressed the following three questions: (1) What are the main community assembly processes in different life stage transitions? (2) Which island attributes influence the assembly process of plant communities? (3) Which mechanisms drive the assembly processes of plant communities in the fragmented landscape?

## Materials and Methods

### Study site

We conducted plant censuses of islands in a man-made reservoir, the Thousand Island Lake (TIL, 29°22’-29°50’ N and 118°34’-119°15’ E), in Zhejiang Province, East China, with the permission of local manager—Xin’an River Development Corporation. The inundation following dam construction on the Xin’an River in 1959 resulted in the isolation of 1078 land-bridge islands (> 0.25 ha) when the water reached its highest level (108 m a.s.l.) [36]. Currently, most of these forested areas (~90%) are mixed forests dominated by Masson pine (*Pinus massoniana*) in the canopy and broad-leaved plants in the sub-canopy and understory.

### Field surveys

**Fig 2:**
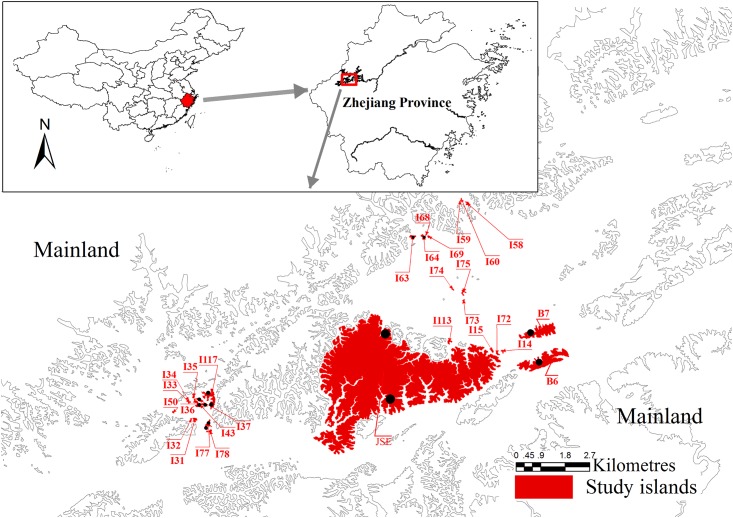
Map of the 29 study islands in the Thousand Island Lake, East China. Black dots indicates the location of transects on the medium and large islands.

We established forest dynamics plots on 29 islands (Fig 2) in TIL in 2009–2010 and set up one 1 ha control plot on the adjacent mainland. These islands were selected to (1) cover a gradient of island areas and isolations, (2) have minimum disturbance, (3) be covered predominantly by Masson pine forests with similar forest structures, and (4) be logistically convenient for fieldwork. Plot establishment and data collection followed the protocol for plot censuses established by the Center for Tropical Forest Science—Forest Global Earth Observatory (http://www.forestgeo.si.edu) network [37,38]. The census of plots on small islands (<1 ha) covered the entire island area. The total areas of sampling plots were 0.5 ha on medium islands (1~5 ha) and 1 ha on large islands (> 5 ha). Analyses conducted as part of previous studies indicated that sampling protocols were sufficient to capture most woody plant species on the large islands [31]. The plots were divided into 5×5m quadrats. The identity, height and diameter at breast height (DBH, breast height = 130cm) of all woody plant individuals with DBH ≧ 1cm were determined/measured [38]. All of the individuals (DBH ≧ 1cm) were then classified as being a tree (DBH≧5cm) or a sapling (1cm ≦ DBH < 5cm). Furthermore, we established 1 × 1m seedling quadrats randomly distributed within each of the plots. The number of seedling quadrats on each island ranged from 4 to 48 depending on the size of the focal island and an area-dependent proportional sampling procedure [39]. In seedling quadrats, all woody seedlings (DBH < 1 cm or height < 130cm) were tagged, identified, and measured. The species were identified with the assistance of taxonomic experts and available literature [40,41].

### Phylogenetic data

The molecular phylogeny for the plant species recorded in the study plots for the analysis of phylogenetic diversity is a pruned version of the one used by Zanne *et al*. [42,43], which included more than 30,000 species of global seed plants. Phylomatic was used to construct the phylogenetic relationships [44] between plant species. We also used the Blomberg’s K [45] to test the phylogenetic niche conservatism of plant functional traits. Several important plant traits including as special leaf area, leaf thickness, chlorophyll concentration and maximal height showed significant phylogenetic signal.

### Island attributes

For each of the 29 islands, area (A, ha) and perimeter length (P, km) were calculated through analysis of maps in ArcGIS 9.3 for periods when the water level is 105 m a.s.l. (generally corresponding to the edge of vegetation on the islands). The shape index (SI) indicates the island shape complexity and was calculated as SI = P/[2×(π×A)^0.5^] [46,47]. SI = 1 when an island is a perfect circle and increases with the more and more irregular and complex shapes. We calculated the isolation (I, km) of each island as the shortest edge-to-edge distance from the focal island to the mainland. To test whether the sampling effect influenced our results, the area of sampling plot was also included in the analysis.

### Species diversity and assembly

We used species richness (number of species) as the index of species diversity (SD). We evaluated if SD is shifting across the different plant life stages. Using the R package “vegan” [48], we computed the total sapling diversity minus total seedling diversity per island and compared these variations to those derived from 1000 randomized communities for each island. We used the species matrix with abundance information in model construction and simulation. The row equals plots/island, and column equals species. The random communities were formed by random sampling from an individual pool (the sum of all plant individuals recorded in all plots) without replacement. In these random models, we maintained abundance per island and total abundance of each species (species frequency) as observed for the whole system. The data of each life stages were simulated and analyzed respectively within independent paths to minimize the potential influence of different sampling sizes and different densities across life stages. The observed and random data were then used to calculate a standardized difference (*Z*_*D*_) of SD in saplings minus that in seedlings as:

$$Z_{D}=\frac{\left(D_{obs.sap}-D_{obs.see}\right)-mean(D_{ran.sap}-D_{ran.see})}{sd(D_{ran.sap}-D_{ran.see})}$$

where *D*_*obs*.*sap*_ and *D*_*obs*.*see*_ are the observed diversity indices of saplings and seedlings, respectively, and *D*_*ran*.*sap*_ and *D*_*ran*.*see*_ are the diversity indices derived from the random communities. Significance was based on one-tailed t-tests. We analyzed the shift of SD between trees and saplings using the same methods as for the shift between saplings and seedlings. Positive values of *Z*_*D*_ indicate increasing standardized SD from the younger life stage to the older stage, corresponding to a species-level overdispersion assembly process. Conversely negative *Z*_*D*_ values indicate a clustering assembly process.

### Phylogenetic diversity and assembly

We used Faith’s PD to assess phylogenetic diversity (PD). Under this metric, PD is the sum of the phylogenetic branch lengths linking together all species present in the focal community [49,50]. We tested the significance of the observed changes in PD across life stages using simulations analogous to those described above for species diversity. Different from the null models used for tests of SD, the null models for PD were randomized according to the independent swap algorithm maintaining the total abundance and the frequency of species occurrences. The *Z*_*D*_ of PD was also calculated as described above for SD and negative and positive values reflect phylogenetic overdispersion and clustering, respectively. Spearman’s rank correlation was used to test the relationship between SD and PD.

All the phylogenetic metrics were calculated by the R packages “picante” [51,52].

### Analyses of diversity shifts

We used a hierarchical regression tree analysis to evaluate the effects of the island attributes linked to habitat fragmentation on the patterns of community assembly between the different life stages. As a non-parametric method, the regression tree is robust to many types of data such as nonlinear relationships and missing values [53]. It is therefore a powerful tool in identifying the key attributes determining shifts of species and phylogenetic diversities. The attributes included in our analyses were island area, isolation, shape index and sampling area. Hierarchical regression tree analyses were conducted using the function {rpart}, and the approximate r-square values of the regression tree were calculated using the function {rsq.rpart} in the R package “rpart” [54].

## Results

### General sampling results

The densities of trees, saplings and seedlings were 1441, 13184 and 244 individuals per hectare, respectively, on the 29 study islands with a total area of 1258.7 ha (S1 Table). And the densities of trees, saplings and seedlings were 1660, 9207 and 5000 individuals per hectare in the mainland plot. The density of seedlings on islands was far less than that on the surrounding mainland. In total, we censused individuals of 92 woody plant species including 88 angiosperms and 4 gymnosperms (S2 Table).

### Changes of species and phylogenetic diversity across life stages

Species diversity (SD) and phylogenetic diversity (PD) differed across the three life stages of plants on islands in TIL. As expected and in accord with previous studies [55–57], there were significant correlations between species diversity and phylogenetic diversity at all three life stages (Tree: *R* = 0.98, *P*<0.001; Sapling: *R* = 0.99, *P*<0.001; Seedling: *R* = 0.90, *P*<0.001) by Spearman’s correlation analysis.

The standardized SD (*Z*_*D*_) in SS decreased on all islands and mainland. However, the standardized SD in ST decreased on 4 islands and mainland, but increased on the other 25 islands. We also found that most plant communities (25 islands) exhibited opposing trends of SD in SS vs. ST comparisons (S3 Table). The standardized PD declined in SS on 17 islands and increased on 10 islands and mainland. The standardized PD declined in ST on 8 islands and increased on 20 islands and the mainland. Opposing trends of PD in SS vs. ST comparisons occurred on 16 islands.

### Effects of island attributes on species and phylogenetic diversity

**Fig 3:**
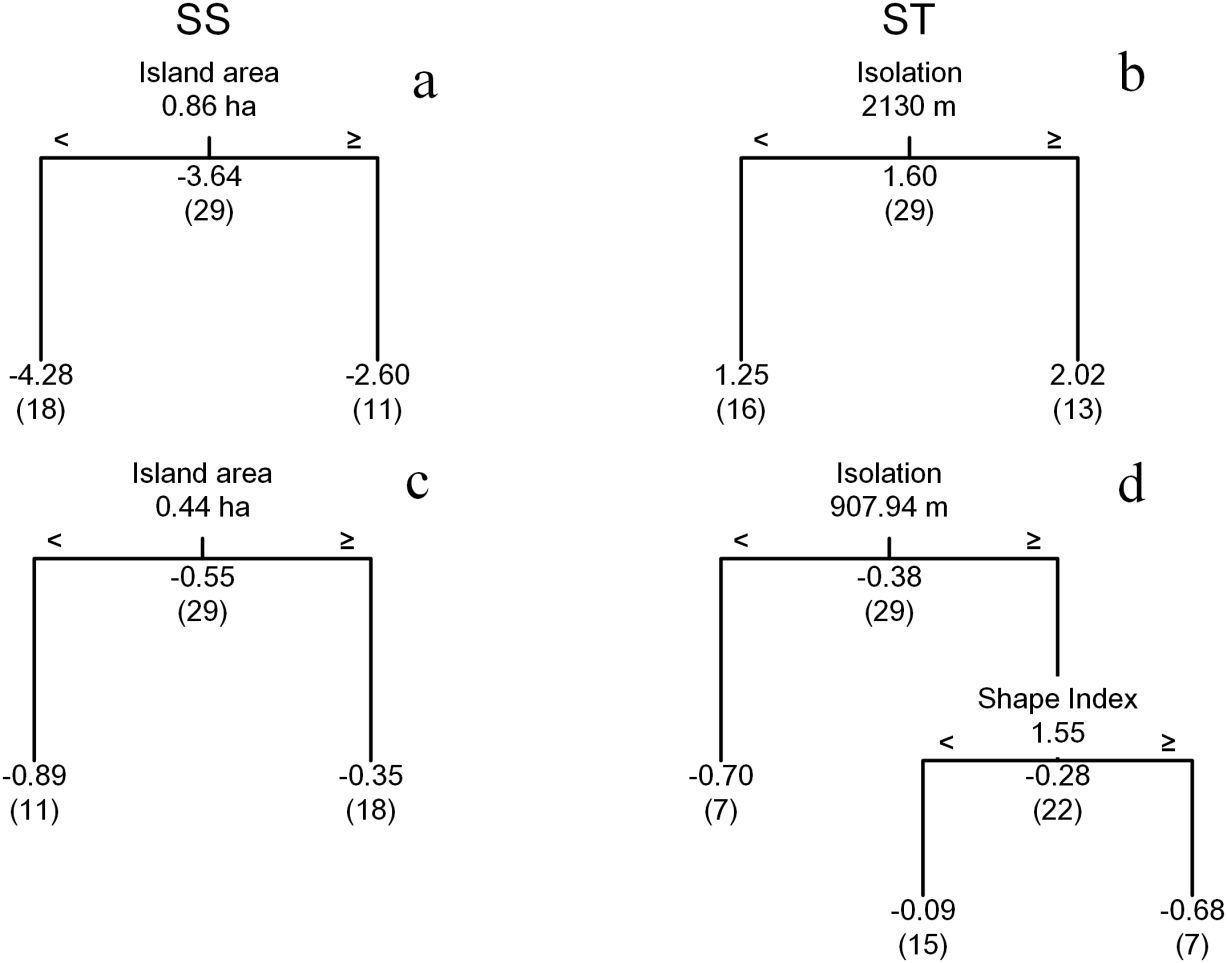
Regression trees for predicting standardized differences of the species (a, b) and phylogenetic (c, d) diversity (SD and PD, respectively) within the SS and ST transitions. The related attributes were island area, isolation, shape index and sampling area.

**Fig 4:**
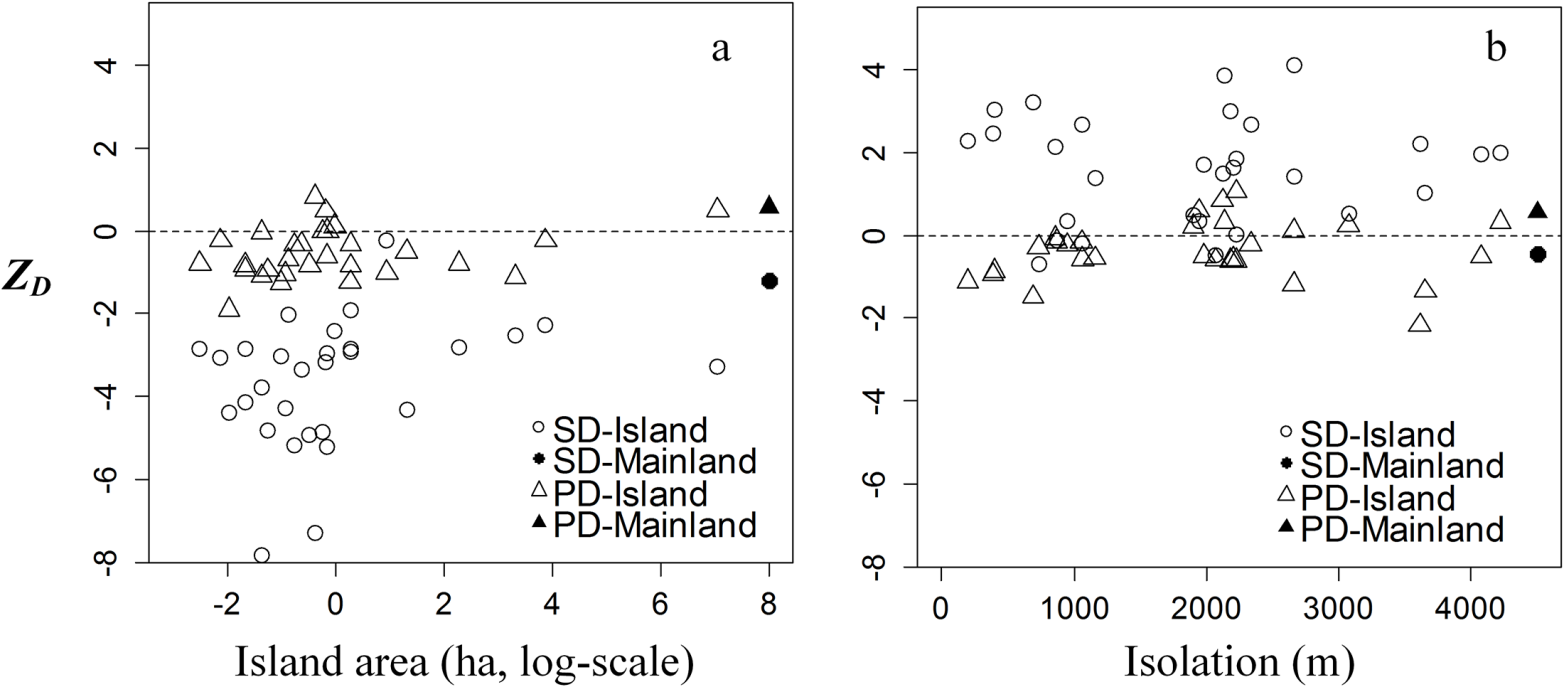
Standardized differences of the species and phylogenetic diversity (SD and PD, respectively) within the SS (a) and ST (b) on 29 study islands and surrounding the mainland.

According to the regression tree analysis, island area is the key island attribute affecting *Z*_*D*_ of SD and PD in the SS transition. Approximate r-square indicated island area explained 28.5% and 9.1% of the variances of SD and PD, respectively, within the SS transition (Fig 3a and 3c). While isolation explained 19.4% and 9.3% of the variances of SD and PD, respectively, during the ST transition (Fig 3b and 3d). We also found the *Z*_*D*_ of SD and PD were both negative on the islands in the SS transition, but that the *Z*_*D*_ of PD were positive on the mainland (Fig 4a). The *Z*_*D*_ of SD and PD on the islands in the ST transition were opposite to those on the mainland (Fig 4b). Sampling area was not a significant factor in the analysis, suggesting that our results are not influenced by sampling effect.

## Discussion

It is widely recognized that mechanisms influencing species co-existence (e.g., environmental filtering, competition, dispersal limitation or neutral process) can work in parallel [58,59], and that the signatures of these mechanisms in terms of over- and under dispersion of phylogenetic or trait-diversity can even cancel other out. In the current study, we found that the assembly process exhibited a non-monotonic pattern with ontogeny. Furthermore, we found that community assembly within some life-stage transitions (SS or ST) even shifted to another pattern along the gradient of island attributes associated with habitat fragmentation. Regardless of life-stage transition, the loss of habitat area was found to be the main ecological factor affecting community assembly [31,32].

On most of the TIL study islands, we found that the changes in SD and PD differed between SS and ST transitions (Fig 4). SD and PD showed clustering on islands, except SD in the ST transition which showed overdispersion. This finding may be due to different ecological processes controlling community assembly processes through the SS and ST transitions. The clustering of PD from juvenile to adult trees was found in rain forest fragments [15], which was considered to reflect the effects of environmental filtering on young trees. Conversely, in other forests, the overdispersion of seedling PD was believed to be due to negative density dependence [2,27]. Swenson *et al*. [60] even found opposing mechanisms controlled the assembly patterns of plant traits related to growth rate, productivity and regeneration, which were respectively linked to the main functions of juvenile and adult cohorts in the same forest. The performance of SD and PD on islands were also different than those on the mainland. On the mainland, SD was clustered and PD was overdispersed. According to the relations between the ecological mechanisms and the shift of SD and PD (Table 1), we considered that environmental filtering or dispersal limitation would be the main driving mechanisms on fragmented islands, while competition drove the community assembly process on the mainland. The difference between islands and the mainland also illustrates that habitat fragmentation can inflate or shift the community assembly processes in plant communities due to smaller habitat area and stronger isolation than the intact forests on mainland. Another important factor is the different floristic composition on these islands which itself is driven by the differential responses of species to habitat loss and fragmentation [30–32]. The rare and common species had different responses and tolerances to disturbance. In our previous study at TIL, we found that the ratio of rare and common species changed across a gradient of habitat fragmentation [30] and that this pattern was caused by differential immigration and colonization of these species.

The results of the regression tree analyses help to distinguish the contribution of environmental filtering and dispersal limitation on community assembly on these islands. Area loss is commonly thought to be the most significant factor affecting biodiversity and community assembly in fragmented landscape, and can in fact mask other effects linked to habitat fragmentation [28]. The result of our analyses show that the shift of SD and PD during SS were strongly associated with island area (Fig 3). Area loss caused the absence of some rare habitat types (especially some resources necessary for rare species) on the islands [61], which would lead a strong effects of environmental filtering on local plant community and decreased rare species diversity on the islands [30]. On the other hand, the effects of environmental filtering only worked in juvenile stages of the plants and appeared to not affect adults.

Isolation also had an important effect by decreasing the connectivity of the fragmented landscape. In this study, isolation was the most important factor in the ST transition (Fig 3b and 3d), but only had a small explanatory power. We have previously found that isolation did not affect SD of vascular plants (including herbaceous and fern species) and birds in TIL [31]. Indeed, isolation only indirectly affected some special groups [31], which was the consequence of selective immigration along the isolation gradient [9]. We speculate that isolation is actually affecting the processes of dispersal limitation. But the strong area effect masked its effect in juvenile stages. The effect of isolation was revealed in the adult stage after the termination of area effect. The unexplained variance meant that other factors not included in this study (e.g., soil condition and animal community composition) may also be affecting the community assembly across plant life stages.

Competition was the main mechanism driving the community assembly on the surrounding mainland. We speculate that the habitat quality of surrounding mainland was poor leading to intense competition for resources. Distinct from the assembly process on islands, plant community assembly on the mainland exhibited a monotonic process across life stages further suggesting that fragmentation changed the plant community assembly [2,14,15,62,63].

## Conclusion

Habitat fragmentation can change ecological patterns, processes, and interactions in plant communities [28,32], including community assembly process [29]. In the Thousand Island Lake region, different assembly processes across plant life stage transitions are exhibited on fragmented islands and the mainland. Our results show that habitat fragmentation modifying landscape configuration area and connectivity can influence community assembly by changing habitat heterogeneity and immigration. A strong but ephemeral effect of environmental filtering mainly drove the assembly process on islands in the juvenile stage of plants. The effect of dispersal limitation was weak but long-term and was revealed in the adult stage after the termination of environmental filtering. Competition was the main driver of plant community assembly in intact forests on the surrounding mainland. These results highlight that besides the internal biotic and abiotic factors within communities, the external factors across habitats and the landscape-scale process are also very important in shaping the community assembly process in fragmented systems [64]. This finding adds to our understanding to community assembly and species coexistence under the circumstance of pervasive and widespread habitat loss and fragmentation.

## Supporting Information

S1 Table Species composition of tree, sapling and seedling on the 29 study islands and mainland. (XLSX)

S2 Table Species list in the study plots on the 29 study islands and surrounding mainland at the Thousand Island Lake. (DOCX)

S3 Table Standard differences of species and phylogenetic diversity across life stages and the summary of island attributes on the 29 study islands and mainland. (DOCX)
