# Supplementary material for: Habitat Fragmentation Drives Plant Community Assembly Processes across Life Stages
Source: PLoS One. 2016 Jul 18;11(7):e0159572. doi: 10.1371/journal.pone.0159572 (PMC4948860; doi:10.1371/journal.pone.0159572)
Supplement: S2 Table — (DOCX) [file pone.0159572.s002.docx]

Table S1 The species list in the study plots on the 29 study islands and surrounding mainland at the Thousand Island Lake

| **Species** | **Code** | **Family** |
| --- | --- | --- |
| *Pistacia chinensis* | Pischi | Anacardiaceae |
| *Rhus chinensis* | Rhuchi | Anacardiaceae |
| *Toxicodendron succedaneum* | Toxsuc | Anacardiaceae |
| *Ilex chinensis* | Ilechi | Aquifoliaceae |
| *Ilex cornuta* | Ilecor | Aquifoliaceae |
| *Ilex rotunda* | Ilerot | Aquifoliaceae |
| *Ilex suaveolens* | Ilesua | Aquifoliaceae |
| *Chimonanthus nitens* | Chinit | Calycanthaceae |
| *Abelia chinensis* | Abechi | Caprifoliaceae |
| *Viburnum dilatatum* | Vibdil | Caprifoliaceae |
| *Viburnum setigerum* | Vibset | Caprifoliaceae |
| *Alangium chinense* | Alachi | Cornaceae |
| *Alangium kurzii* | Alakur | Cornaceae |
| *Cunninghamia lanceolata* | Cunlan | Cupressaceae |
| *Cupressus funebris* | Cupfun | Cupressaceae |
| *Juniperus formosana* | Junfor | Cupressaceae |
| *Diospyros glaucifolia* | Diogla | Ebenaceae |
| *Diospyros kaki* | Diosyl | Ebenaceae |
| *Lyonia ovalifolia* | Lyoova | Ericaceae |
| *Rhododendron mariesii* | Rhomar | Ericaceae |
| *Rhododendron molle* | Rhomol | Ericaceae |
| *Rhododendron ovatum* | Rhoova | Ericaceae |
| *Rhododendron simsii* | Rhosim | Ericaceae |
| *Vaccinium bracteatum* | Vacbra | Ericaceae |
| *Vaccinium carlesii* | Vaccar | Ericaceae |
| *Vaccinium mandarinorum* | Vacman | Ericaceae |
| *Glochidion puberum* | Glopub | Euphorbiaceae |
| *Mallotus apeltus* | Malape | Euphorbiaceae |
| *Mallotus japonicus* | Maljap | Euphorbiaceae |
| *Sapium seibiferum* | Sapsei | Euphorbiaceae |
| *Vernicia montana* | Vermon | Euphorbiaceae |
| *Albizia kalkora* | Albkal | Fabaceae |
| *Dalbergia hupeana* | Dalhup | Fabaceae |
| *Indigofera pseudotinctoria* | Indpse | Fabaceae |
| *Lespedeza bicolor* | Lesbic | Fabaceae |
| *Castanopsis sclerophylla* | Casscl | Fagaceae |
| *Cyclobalanopsis glauca* | Cycgla | Fagaceae |
| *Lithocarpus glaber* | Litgla | Fagaceae |
| *Quercus acutissima* | Queacu | Fagaceae |
| *Quercus fabri* | Quefab | Fagaceae |
| *Quercus serrata* | Queser | Fagaceae |
| *Liquidambar formosana* | Liqfor | Hamamelidaceae |
| *Loropetalum chinense* | Lorchi | Hamamelidaceae |
| *Platycarya strobilacea* | Plastr | Juglandaceae |
| *Cinnamomum camphora* | Cincam | Lauraceae |
| *Lindera aggregata* | Linagg | Lauraceae |
| *Lindera glauca* | Lingla | Lauraceae |
| *Lindera reflexa* | Linref | Lauraceae |
| *Litsea coreana* | Litcor | Lauraceae |
| *Litsea cubeba* | Litcub | Lauraceae |
| *Sassafras tzumu* | Sastzu | Lauraceae |
| *Buddleja lindleyana* | Budlin | Loganiaceae |
| *Magnolia cylindrica* | Magcyl | Magnoliaceae |
| *Broussonetia kazinoki* | Brokaz | Moraceae |
| *Myrica rubra* | Myrrub | Myricaceae |
| *Syzygium buxifolium* | Syzbux | Myrtaceae |
| *Chionanthus retusus* | Chiret | Oleaceae |
| *Fraxinus insularis* | Frains | Oleaceae |
| *Pinus massoniana* | Pinmas | Pinaceae |
| *Pleioblastus amarus* | Pleama | Poaceae |
| *Punica granatum* | Pungra | Punicaceae |
| *Rhamnus crenatus* | Rhacre | Rhamnaceae |
| *Photinia parvifolia* | Phopar | Rosaceae |
| *Photinia serrulata* | Phoser | Rosaceae |
| *Pyrus calleryana* | Pyrcal | Rosaceae |
| *Raphiolepis indica* | Rapind | Rosaceae |
| *Gardenia jasminoides* | Garjas | Rubiaceae |
| *Serissa japonica* | Serjap | Rubiaceae |
| *Poncirus trifoliata* | Pontri | Rutaceae |
| *Xylosma racemosum* | Xylrac | Salicaceae |
| *Itea oblonga* | Iteobl | Saxifragaceae |
| *Euscaphis japonica* | Eusjap | Staphyleaceae |
| *Alniphyllum fortunei* | Alnfor | Styracaceae |
| *Styrax confusus* | Stycon | Styracaceae |
| *Styrax dasyanthus* | Stydas | Styracaceae |
| *Styrax faberi* | Styfab | Styracaceae |
| *Styrax odoratissimus* | Styodo | Styracaceae |
| *Symplocos paniculata* | Sympan | Symplocaceae |
| *Symplocos stellaris* | Symste | Symplocaceae |
| *Symplocos sumuntia* | Symsum | Symplocaceae |
| *Camellia fraterna* | Camfra | Theaceae |
| *Eurya japonica* | Eurjap | Theaceae |
| *Eurya muricata* | Eurmur | Theaceae |
| *Schima superba* | Schsup | Theaceae |
| *Ternstroemia gymnanthera* | Tergym | Theaceae |
| *Wikstroemia monnula* | Wikmon | Thymelaeaceae |
| *Trema cannabina* | Trecan | Ulmaceae |
| *Ulmus parvifolia* | Ulmpar | Ulmaceae |
| *Clerodendrum cyrtophyllum* | Clecyr | Verbenaceae |
| *Clerodendrum kaichianum* | Clekai | Verbenaceae |
| *Premna microphylla* | Premic | Verbenaceae |
| *Vitex negundo* | Vitneg | Verbenaceae |
