# Supplementary material for: Habitat Fragmentation Drives Plant Community Assembly Processes across Life Stages
Source: PLoS One. 2016 Jul 18;11(7):e0159572. doi: 10.1371/journal.pone.0159572 (PMC4948860; doi:10.1371/journal.pone.0159572)
Supplement: S3 Table — (DOCX) [file pone.0159572.s003.docx]

Table S2 Standard differences of species and phylogenetic diversity across life stages and the summary of island attributes on the 29 study islands and mainland. SD: *Z_D_* of species diversity; PD: *Z_D_* of phylogenetic diversity.

| Island | SS | | ST | | Area (ha) | Shape index | Isolation (m) |
| --- | --- | --- | --- | --- | --- | --- | --- |
|  | SD | PD | SD | PD |  |  |  |
| B6 | -2.28 | -0.24 | 2.68 | -0.57 | 47.98 | 3.06 | 1066.10 |
| B7 | -2.52 | -1.11 | 1.38 | -0.55 | 27.49 | 3.02 | 1158.87 |
| I113 | -2.42 | 0.1 | 1.98 | 0.33 | 0.99 | 1.54 | 4217.10 |
| I117 | -2.83 | -0.79 | 0.47 | 0.21 | 9.79 | 3.19 | 1901.72 |
| I14 | -5.19 | -0.35 | 1.71 | -0.49 | 0.46 | 1.42 | 1982.69 |
| I15 | -4.93 | -0.82 | 2.69 | -0.23 | 0.62 | 1.27 | 2333.60 |
| I31 | -4.87 | -0.02 | 1.42 | -1.18 | 0.79 | 1.28 | 2657.77 |
| I32 | -7.82 | -1.1 | 4.12 | 0.1 | 0.25 | 1.21 | 2658.07 |
| I33 | -3.04 | -1.26 | 0.02 | 1.05 | 0.36 | 1.28 | 2227.08 |
| I34 | -3.06 | -0.24 | -0.47 | -0.59 | 0.12 | 1.20 | 2073.07 |
| I35 | -3.37 | -0.33 | 0.33 | 0.59 | 0.53 | 1.44 | 1939.94 |
| I36 | -2.87 | -0.83 | 3.86 | 0.32 | 0.19 | 1.11 | 2137.68 |
| I37 | -2.86 | -0.33 | 1.5 | 0.84 | 1.32 | 1.43 | 2121.37 |
| I43 | -4.33 | -0.51 | 1.85 | -0.61 | 3.70 | 2.00 | 2225.45 |
| I50 | -4.82 | -0.95 | 0.52 | 0.23 | 0.29 | 1.07 | 3073.21 |
| I58 | -3.18 | 0.48 | 3.22 | -1.49 | 0.84 | 1.39 | 690.03 |
| I59 | -4.14 | -0.92 | 3.02 | -0.86 | 0.19 | 1.44 | 398.18 |
| I60 | -4.38 | -1.9 | 2.45 | -0.95 | 0.14 | 1.26 | 387.10 |
| I63 | -1.91 | -0.85 | -0.71 | -0.28 | 1.33 | 1.48 | 730.95 |
| I64 | -2.91 | -1.24 | -0.11 | -0.14 | 1.31 | 1.39 | 868.22 |
| I68 | -2.84 | -0.79 | 0.35 | -0.23 | 0.08 | 1.03 | 947.67 |
| I69 | -2.01 | -0.7 | -0.19 | -0.17 | 0.42 | 1.07 | 1056.16 |
| I72 | -7.29 | 0.82 | 2.28 | -1.1 | 0.69 | 1.44 | 200.31 |
| I73 | -3.78 | -0.04 | 1.02 | -1.34 | 0.25 | 1.16 | 3650.55 |
| I74 | -4.27 | -1.04 | 1.97 | -0.53 | 0.39 | 1.56 | 4075.04 |
| I75 | -2.97 | 0.01 | 2.22 | -2.17 | 0.86 | 2.18 | 3609.61 |
| I77 | -0.25 | -1 | 1.63 | -0.58 | 2.56 | 2.14 | 2199.38 |
| I78 | -5.22 | -0.62 | 3 | -0.6 | 0.85 | 1.49 | 2184.55 |
| JSE | -3.28 | 0.51 | 2.14 | -0.08 | 1153.88 | 8.46 | 861.00 |
| Mainland | -1.2 | 0.58 | -0.43 | 0.55 | - | - | - |
